# Comparing machine learning screening approaches using clinical data and cytokine profiles for COVID-19 in resource-limited and resource-abundant settings

**DOI:** 10.1038/s41598-024-63707-3

**Published:** 2024-06-28

**Authors:** Hooman H. Rashidi, Aamer Ikram, Luke T. Dang, Adnan Bashir, Tanzeel Zohra, Amna Ali, Hamza Tanvir, Mohammad Mudassar, Resmi Ravindran, Nasim Akhtar, Rana I. Sikandar, Mohammed Umer, Naeem Akhter, Rafi Butt, Brandon D. Fennell, Imran H. Khan

**Affiliations:** 1 Computational Pathology and AI Center of Excellence (CPACE), University of Pittsburgh Medical Center, and University of Pittsburgh School of Medicine, Pittsburgh, USA; 2 Department of Pathology and Laboratory Medicine, University of California, 4400 V Street, DavisSacramento, CA 95817 USA; 3 National Institutes of Health, Islamabad, Pakistan; 4 Health Information Systems Program (HISP), Islamabad, Pakistan; 5 Pakistan Institute of Medical Sciences, Islamabad, Pakistan; 6 Rawalpindi Medical University-Rawalpindi, Rawalpindi, Pakistan; 7 Isolation Hospital and Infectious Treatment Centre, Islamabad, Pakistan; 8 Department of Medicine, University of California, San Francisco, USA

**Keywords:** COVID-19, Cytokines, Chemokines, Binary classifier, Machine learning, FGF basic (FGF2), Eotaxin (CCL11), G-CSF (CSF3), GM-CSF (CSF2), IFN-γ (IFNG), IL-1β (IL1B), IL-1ra (IL1RN), IL-1α (IL1A), IL-2Rα (IL2RA), IL3, IL-12 (p40) (IL12B), IL16, IL2, IL4, IL5, IL6, IL7, IL8 (CXCL8), IL9, GRO-α (CXCL1), HGF, IFN-α2 (IFNA2), LIF, MCP-3 (CCL7), IL10, IL-12 (p70) (IL12A), IL13, IL15, IL17A, IP-10 (CXCL10), MCP-1 (MCAF) (CCL2), MIG (CXCL9), β-NGF (NGF), SCF (KITLG), SCGF-β (CLEC11A), SDF-1α (CXCL12), MIP-1α (CCL3), MIP-1β (CCL4), PDGF-BB (PDGFB), RANTES (CCL5), TNF-α, VEGF (VEGFA), CTACK (CCL27), MIF, TRAIL (TNFSF10), IL-18 (IL18), M-CSF (CSF1), TNF-β (LTA), Predictive markers, Translational research, Laboratory techniques and procedures, Software, Information technology

## Abstract

Accurate screening of COVID-19 infection status for symptomatic patients is a critical public health task. Although molecular and antigen tests now exist for COVID-19, in resource-limited settings, screening tests are often not available. Furthermore, during the early stages of the pandemic tests were not available in any capacity. We utilized an automated machine learning (ML) approach to train and evaluate thousands of models on a clinical dataset consisting of commonly available clinical and laboratory data, along with cytokine profiles for patients (n = 150). These models were then further tested for generalizability on an out-of-sample secondary dataset (n = 120). We were able to develop a ML model for rapid and reliable screening of patients as COVID-19 positive or negative using three approaches: commonly available clinical and laboratory data, a cytokine profile, and a combination of the common data and cytokine profile. Of the tens of thousands of models automatically tested for the three approaches, all three approaches demonstrated > 92% sensitivity and > 88 specificity while our highest performing model achieved 95.6% sensitivity and 98.1% specificity. These models represent a potential effective deployable solution for COVID-19 status classification for symptomatic patients in resource-limited settings and provide proof-of-concept for rapid development of screening tools for novel emerging infectious diseases.

## Introduction

Since its emergence on the global stage in early 2020, the novel coronavirus SARS-CoV-2 (COVID-19) has exerted a profound impact on human health^1–3^. In low resource settings, utilization of more commonly available clinical and laboratory data to stratify patients would be ideal since basic clinical history taking, vital signs, physical examination, and routine laboratory data (from chemistry and cell analyzers) can yield a multitude of useful data suitable for machine learning^4–8^. Indeed, in the infectious disease setting, such methods have been previously applied towards datasets in tuberculosis^9–13^. In addition, in more resource abundant settings, the quantification of the vast array of cytokines and chemokines^14^ has provided utility in both the detection and stratification of disease severity of COVID-19 infected patients^15–18^.

We decided to explore how these two sources of information, when coupled with machine learning models could perform in the detection of COVID-19. Traditional ML development relies on the experience of highly trained data scientists to match algorithms to a given problem (which may introduce selection bias or human preferences into the process)^19^. In contrast, for this study we utilized an automated ML platform (MILO: Machine Intelligence Learning Optimizer) which combines supervised and unsupervised ML methods (including various algorithm types, feature selectors, and feature transformers) to enable empiric (without human bias) and high-throughput generation of classifier models (over 1000 unique pipelines yielding hundreds of thousands of ML models). All solutions (> 300,000 potential ML models) are attempted empirically, without human bias, and evaluated based on performance metrics in an out-of-sample dataset. This platform has previously been applied to develop clinically relevant classifiers for acute kidney injury to predict sepsis in burn patients^20–23^, to predict delayed graft function in renal allografts^24^, to predict COVID-19 infection from MALDI-TOF-MS data^25^, and to predict tuberculosis status from multiplex serologic data from an endemic setting^26^. These prior studies demonstrate the utility of MILO in a wide variety of clinical applications to provide classifiers with improved sensitivity and specificity^27–30^.

In this study, we sought to apply the aforementioned automated ML approach to the critical problem of COVID-19 status prediction utilizing clinical, demographic, and routine laboratory data as available in a resource-limited setting as well as cytokine and chemokine serologic assays to establish both immediately applicable clinical decision models as well as explore possible pathways for the rapid development and deployment of screening and diagnostic tools for future epidemics and pandemics.

## Results

This study aimed to develop and identify an optimal binary classifier for COVID-19 status of symptomatic patients using a tiered approach comparing common clinical and laboratory findings (Approach 1), a cytokine fingerprint (Approach 2), or a combination of both (Approach 3), as shown in Table 1. For each approach, the MILO platform was utilized to generate over 300,000 models from a diverse set of algorithms (Neural Network [NN], Logistic Regression [LR], Naive Bayes [NB], k-Nearest Neighbors [k-NN], Support Vector Machine [SVM], Random Forest [RF], Extreme Gradient Boosting [XGBoost]) while simultaneously varying additional elements of the ML pipeline including hyperparameters, data scaling, feature selection, etc., by training on dataset A^26^. These models were subsequently validated on a secondary dataset (B), which the models were naïve to and were never previously trained on, to provide robust and true performance metrics for evaluation of the models^26^. In this paradigm, two separate performance measures are captured for model comparison which further reduces the likelihood of overfitting in the selected model.

**Table 1:** Performance metrics for best classification models for each approach.

Statistic	Approach 1: lab only^1^		Approach 2: cytokine only^2^		Approach 3: combined^3^	
	Value	95% CI	Value	95% CI	Value	95% CI
Accuracy	0.958	0.905–0.986	0.908	0.842–0.953	0.967	0.917–0.991
ROC AUC	0.985	0.841–1	0.936	0.842–1	0.970	0.755–1
Sensitivity	0.927	0.837–0.976	0.927	0.837–0.976	0.956	0.876–0.991
Specificity	1	0.932–1	0.885	0.766–0.957	0.981	0.897–1
PPV	1	0.920–1^a^	0.913	0.831–0.957	0.985	0.903–0.998
NPV	0.912	0.817–0.960	0.902	0.794–0.956	0.944	0.849–0.981

GBM, gradient boosting machine; LR, logistic regression; RFI, random forest importance.

^1^GBM with all features.

^2^LR with 50% of features via select percentile.

^3^GBM with 25% of features via RFI.

^a^CI determined after initial analysis using Wilson score interval.

When developing models with the end goal of clinical deployment, it is ideal to reduce the number of input features so that only features which increase the overall signal-to-noise ratio are utilized. This also ensures the model is executable in a larger proportion of clinical cases, rather than having missing data elements (features) which preclude utilization. Therefore, the identification of such a model is a pragmatic objective, as well as illustrating the utility of the automated ML approach which utilizes a high-throughput approach to evaluate a range of feature sets and derivative models. Indeed, as illustrated in Supplemental Table 1, the parallel workflow of MILO generates a range of functional models with acceptable performance metrics which can be selected based on the intended clinical application (screening versus confirmatory test, etc.). In this case, many of the top models are derived from random forest algorithms, although neural network models with good performance are seen as well. However, it cannot be known ahead of time which algorithm will perform best on a given dataset, which is why parallel testing provides empiric assessment of the optimal models which might otherwise not be noted. Accordingly, we report here the development of a model which enables a prediction to be made with only readily discernable clinical features, as well as limited laboratory data which could be obtained even in resource limited settings from a complete blood count and basic metabolic panel).

From the automated model testing process, Approach 1, using only clinical data, revealed an optimal model using Gradient Boosting Machine with all features achieving a Sensitivity of 92.7% (95CI 83.7–97.6%) and Specificity of 100% (95CI 93.2–100%). Approach 2 using the cytokine analysis found an optimal model of Logistic Regression using 50% of features via Select Percentile achieving a Sensitivity of 92.7% (95CI 83.7–97.6%) and Specificity of 88.5% (95CI 76.6–95.7%). The selected cytokines/chemokines were IL-1ra, IL-2R alpha, IL-4, IL-6, IL-7, IL-10, IL-12 (p40), IL-13, IL-17, MCP-1, MCP-3, MIG, IFN-a2, IFN-g, TNF-b, GM-CSF, M-CSF, LIF, PDFB-BB, IP-10, HGF, SCF, CTACK, SCFG-b.

Approach 3, using the combined clinical data and cytokine analysis, found an optimal model of Gradient Boosting Machine with 25% of features selected via Random Forest Importance achieving a Sensitivity of 95.6% (95CI 87.6–99.1%) and a Specificity of 98.1% (95CI 89.7–100%). The 25% of clinical data features selected included heart rate, diagnosed diabetes, presence of ARDS, need for supplemental oxygen, room air O2 saturation, Chest X-ray results, white blood cell and lymphocyte counts, hemoglobin, platelet count, potassium, and ALT. Additional utilized features from the cytokines/chemokine dataset included IL-1ra, IL-6, IL-10, IL-18, IP-10, MCP-1, MCP-3, MIG, G-CSF, HGF, TRAIL, RANTES, and Eotaxin. Of note, IL-18, G-CSF, TRAIL, RANTES, and Eotaxin were selected for Approach 3, but not utilized in Approach 2.

**Figure 1:**
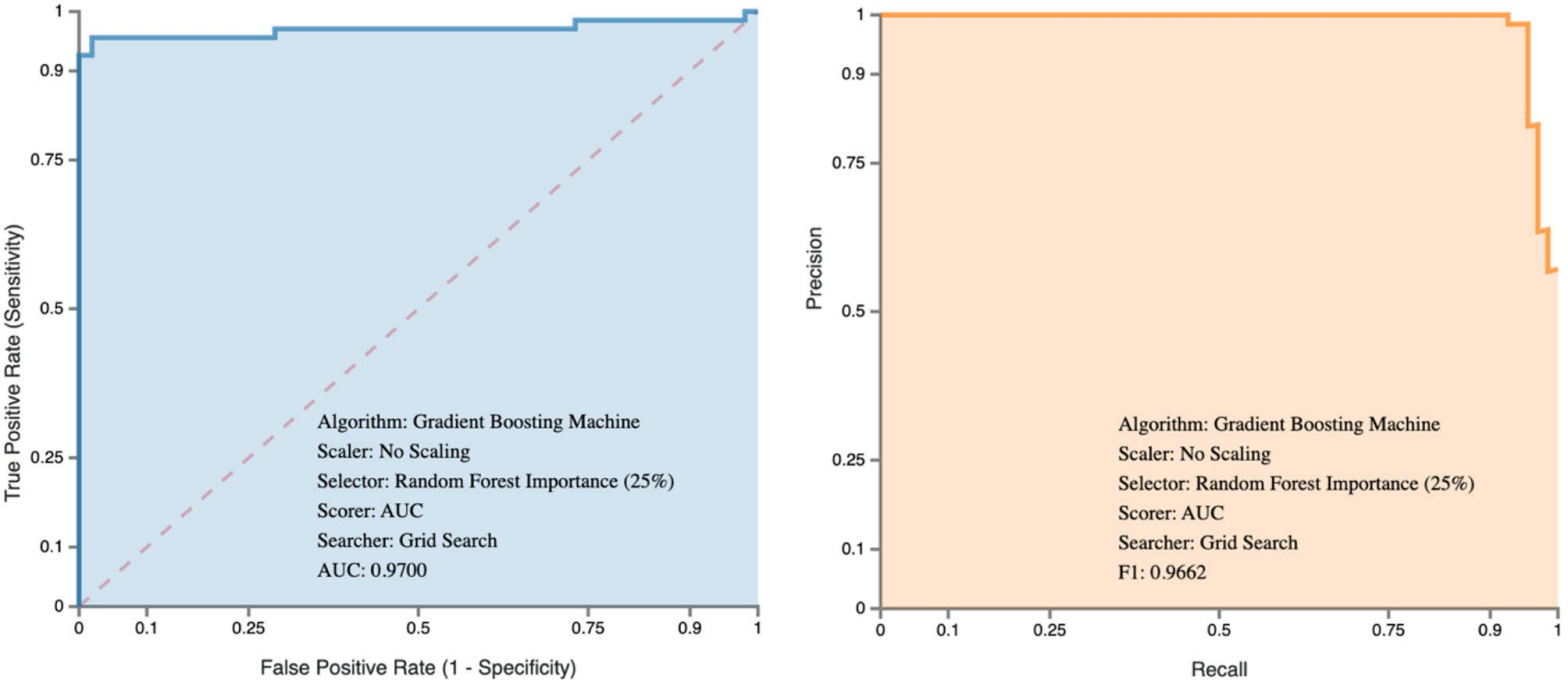
ROC AUC and precision recall curves for approach 3.

Additional performance metrics including PPV, NPV, Accuracy, and ROC AUC are summarized in Table 1. Notably, all three models demonstrated ROC AUC values greater than 93%. ROC AUC curves for our highest-performing model from Approach 3 are shown in Fig. 1. The ROC AUC curves for Approaches 1 and 2 are available in the Supplemental Materials (Supplemental Fig. 1).

The additional twenty highest-performing models for each Approach as determined by ROC AUC are provided in the Supplemental Materials for further review (Supplemental Table 1). The parallel workflow of MILO generates a range of functional models with acceptable performance metrics which can be selected based on the intended clinical application (screening versus confirmatory test, etc.).

## Discussion

The rapid development of predictive and algorithmic models which can integrate readily available and diverse clinical and laboratory data to provide actionable interpretations is an important real-world implication of recent progress in automated machine learning methods. Here, we demonstrate how such models can be developed through such a development pipeline with relatively minimal intervention by a ML domain expert. The performance of these models is notable, especially taking into consideration the range of possible clinical and laboratory data utilized^19–23,25,26^.

These various models also demonstrate a utility in a variety of clinical scenarios with broad applicability for future scenarios in which we face the uncertainty of a new epidemic or pandemic requiring screening/diagnostic testing. In approach 1, commonly available labs and standard clinical information accessible in a range of both resource limited and resource abundant areas allows for the rapid deployment of a clinical prediction tool. It provides real world utility in triaging patients to reduce disease spread, risk-stratifying patients for clinical intervention, and in clinical settings in which such molecular testing is simply not available^19–23,25,26^.

In approach 2, while new antigen POC or serum-based tests specific to a new infectious process are being developed, utilization of a cytokine/chemokine fingerprint derived from a general chemokine/cytokine serum panel allows for screening or diagnostic testing from serum samples without the overhead of gathering the full panel of clinical data. Finally, the combined approach 3 also demonstrates excellent performance metrics when the full range of serologic and clinical information is available from both approaches 1 and 2.

A strength of empirical model development is the discovery of relationships between features which would otherwise elude manual and first-order analysis. Models may not utilize features which have strong correlation to the target if that information is redundant or encoded in alternative features, alternatively, features with obscure correlations may be included because of their value in second or third order relationships through feature engineering, etc. Features with strong correlations on a one-to-one basis may also not display great specificity or may not contribute unique information. Additionally, other features with less strong correlations on a one-to-one basis to the target variable may add unique information, which is otherwise missing, and in combination, the synergistic effect of multiple weakly correlated features may enable the development of a much more powerful model than would be expected. Therefore, although some conclusions may be drawn as to the importance of individual features based on their inclusion in a model, ultimately it is the empiric out-of-sample performance of the models which best reveals the specific combination of features may provide the most reliable binary interpretation of input data.

A follow-up study with additional patients from various institutions and regions would enable validation of these developed models in a more diverse set of patient populations, further increasing confidence in the robustness of the developed in silico models. In addition, these larger datasets may enable further model development utilizing distinct feature sets with even fewer dependencies (features), to improve ease of clinical deployment. Furthermore, a follow up study examining the ability of such an approach to stratify COVID-19 positive patients as high or low risk for ICU admission would be of great clinical relevance. In addition to work needed to further validate the robustness and generalizability of these models to data from various settings, further work is also needed to improve the ease of utilization by clinicians in resource-limited settings that will also increase the impact of such ML developed models in clinical practice. For example, the MILO platform allows for easy deployment of models via a simple user interface as could be accessed via a standard smartphone. Additional work to study the effect of deploying such models in the background of an EMR to alert clinicians to patients with a higher risk of COVID infection based on ubiquitous data within the patient chart would be of interest to optimize clinical management of such patients, and would have broad applicability to other emerging pandemics as well, since rapid model development and deployment using such common clinical data can occur at a pace expected to outmatch specific molecular or protein-based assay development. Indeed, similar iterative efforts could be used to monitor the performance of models in prediction of COVID-19 status as the virus continues to evolve, since variant strains may have evolving clinical manifestations (changing clinical severity, etc.).

Regarding implementation, once a model is developed and validated, the MILO-ML framework provides a pipeline to export the machine learning model using a joblib file (a standard model format) which can be integrated into existing health system machine learning infrastructure. If such infrastructure is not available, the framework also allows for a web-based form accessible from any browser-enabled device such as a smartphone allowing input of clinical data to assess COVID-19 infection risk for individual cases. This approach would be more feasible in Approach 1 where basic clinical data can be inputted. We recommend programmatic integration with an EMR with the use of the joblib file for Approaches 2 and 3 to allow automated inputting of the measured laboratory. For isolated uses of the model, no protected health information (PHI) is required to operate the model for one-off predictions. For integrated approaches, the existing data governance and PHI management protocols will be able to be invoked to ensure the safety and security of patient information. The overall cost of deploymentis variable. The existing models can be run on most Linux, Windows, or macOS computers requiring no additional infrastructure. The primary cost will be the labor and expertise required to meet an institution’s data governance requirements.

The models developed (namely Approach 1) in this study have significant advantages in resource-limited settings, in that only easily obtained clinical data and laboratory data which is restricted to ubiquitous tests (complete blood count and chemistry) is required for model inputs. Therefore, these models could be immediately deployed, including future pandemics, even in regions without new and advanced laboratory facilities. It is important to note even in countries where resources are typically not limited, during times of crisis, such testing may become limited in availability in all settings, as was seen during the peak of the COVID-19 pandemic. Therefore, the rapid development of such models is of great relevance to providing tools to clinicians and public health personnel in order to provide appropriate clinical care and resources to individual patients and at a public health level. Although further generalization testing studies is absolutely required for these models before deployment, this study represents an important proof of concept for the development of algorithmic models using automated machine learning techniques in the infectious disease setting. Further work to validate such approaches will increase readiness for future pandemics, or for recurrent surges in COVID-19 as may occur with the evolution of new variants. The society-wide impact of recent public health crises has served to illustrate the importance of innovative approaches which leverage newly developed technologies such as auto-ML for application in the healthcare domain. Importantly, the broad applicability of these methods means that continuing efforts to optimize rapid model development and deployment will increase readiness for future events, regardless of the specific etiology.

## Methods

### Ethical approval

Approval for the study was obtained from the Institutional Review Board (IRB) of the National Institutes of Health (NIH, Islamabad). Blood samples were drawn from all the patients on the first day of admission after informed written and verbal consent from the patient or guardian, between May 2020 to February 2022. A pre-tested questionnaire was used to document all the clinical and laboratory information. A unique laboratory ID was given to each de-identified blood sample. The aforementioned IRB approval, informed consent, and de-identified sample are in compliance with the guidelines and regulations required by Scientific Reports publishing policy.

### Study design and population

Blood samples were collected in EDTA tubes. Plasma was separated from the blood and stored at − 80 °C until use^15^. Data from 270 symptomatic patients was collected based on three or more of the following signs or symptoms: fever, cough, generalized weakness or fatigue, headache, myalgia, sore throat, coryza (inflammation of the nose), dyspnea, anorexia/nausea/vomiting, diarrhea, or altered mental status, in addition to COVID-19 RT-PCR status. Data for 50 features were collected: age, gender, height, heart rate, respiratory rate, systolic blood pressure, diastolic blood pressure, temperature, weight, hypertension, hospitalization within 90 days, weight loss, chronic lung disease, diabetes, cardiovascular disease, chronic renal disease, taking ACE or ARB medication, NSAID use, cancer family history, diabetes family history, heart disease family history, history of BCG vaccine, history of polio vaccine, current smoking history, past smoking history, shortness of breath, cough, sore throat, body aches, sputum production, nasal congestion, fatigue, chest pain, diarrhea/loose stool, vomiting, abdominal pain, headache, chills, confusion, requirement for O_2_ nasal cannula, O_2_ saturation, alanine transaminase (ALT), lymphocyte count, creatinine, hemoglobin, platelet count, white blood cell count (WBC), sodium (Na), potassium(K), and wheezing. The majority of these features were quantitative, however some of the clinical features were binary in nature. Full tables of descriptive statistics for each of the features is provided in Supplemental Tables 2 and 3.

### Multiplex cytokine/chemokine assay

Multiplex kits for measuring cytokines, chemokines, and growth factors (Cat#12007283), for use on the Luminex platform (Luminex Corp., Austin, TX, USA), were obtained from Bio-Rad, Hercules, CA. Assays were performed according to the manufacturer’s instructions. There were 48 immune molecules/analytes (cytokines/chemokines) in the assay kit that included: FGF basic (FGF2), Eotaxin (CCL11), G-CSF (CSF3), GM-CSF (CSF2), IFN-γ (IFNG), IL-1β (IL1B), IL-1ra (IL1RN), IL-1α (IL1A), IL-2Rα (IL2RA), IL3, IL-12 (p40) (IL12B), IL16, IL2, IL4, IL5, IL6, IL7, IL8 (CXCL8), IL9, GRO-α (CXCL1), HGF, IFN-α2 (IFNA2), LIF, MCP-3 (CCL7), IL10, IL-12 (p70) (IL12A), IL13, IL15, IL17A, IP-10 (CXCL10), MCP-1 (MCAF) (CCL2), MIG (CXCL9), β-NGF (NGF), SCF (KITLG), SCGF-β (CLEC11A), SDF-1α (CXCL12), MIP-1α (CCL3), MIP-1β (CCL4), PDGF-BB (PDGFB), RANTES (CCL5), TNF-α, VEGF (VEGFA), CTACK (CCL27), MIF, TRAIL (TNFSF10), IL-18 (IL18), M-CSF (CSF1), and TNF-β (LTA). The concentration (pg/mL) of each cytokine/chemokine in the multiplex panels was measured based on a 7-point standard curve using xPONENT 4.3 software (Luminex, TX, USA)^16^.

### COVID-19 RT-PCR

Oropharyngeal swab samples were taken and extracted, per manufacturer instructions, on the KingFisher™ Flex Purification System (ThermoFisher Scientific, USA) using the MagMAX Viral/Pathogen Nucleic Acid Isolation kit (ThermoFisher Scientific, USA). The RT-PCR testing was performed on Applied biosystem 7500 Real-Time PCR system (Thermo Fisher Scientific, USA) using Genesig® COVID-19 2G RealTime CE-IVD PCR Assay (Primerdesign Ltd, UK), which targets the ORF1ab and S gene of SARS-CoV-2.

### Design and application of the automated machine learning platform, MILO (Machine Intelligence Learning Optimizer) to the training dataset

The dependent variable for the patient population was COVID-19 positivity as a binary value (1 = COVID positive, 0 = COVID negative). All feature values were collected for all cases used in this study. No imputation of values was utilized. The data was scaled prior to segmentation to ensure equal weights between features during training. The complete raw dataset was then segmented into separate datasets, A and B, using random sampling without replacement (Table 2). Dataset A was used for training and initial validation. The sample was created such that the two classes of COVID positive (n = 75) and negative (n = 75) were perfectly balanced to ensure no bias was introduced during the training process that would skew performance metrics. Dataset A was then used in a ten-fold cross-validation approach within the MILO-ML platform (as detailed below). Dataset B (n = 120) remained completely isolated from the model training/development process only to be used for the evaluation of model performance^26^.

**Table 2:** Training and generalization testing dataset characteristics.

Dataset name	Dataset type	Dataset composition
Dataset A	Training	Total n = 150 COVID-19 Positive n = 75 COVID-19 Negative n = 75
Dataset B	Generalization testing	Total n = 120 COVID-19 Positive n = 68 COVID-19 Negative n = 52

**Figure 2:**
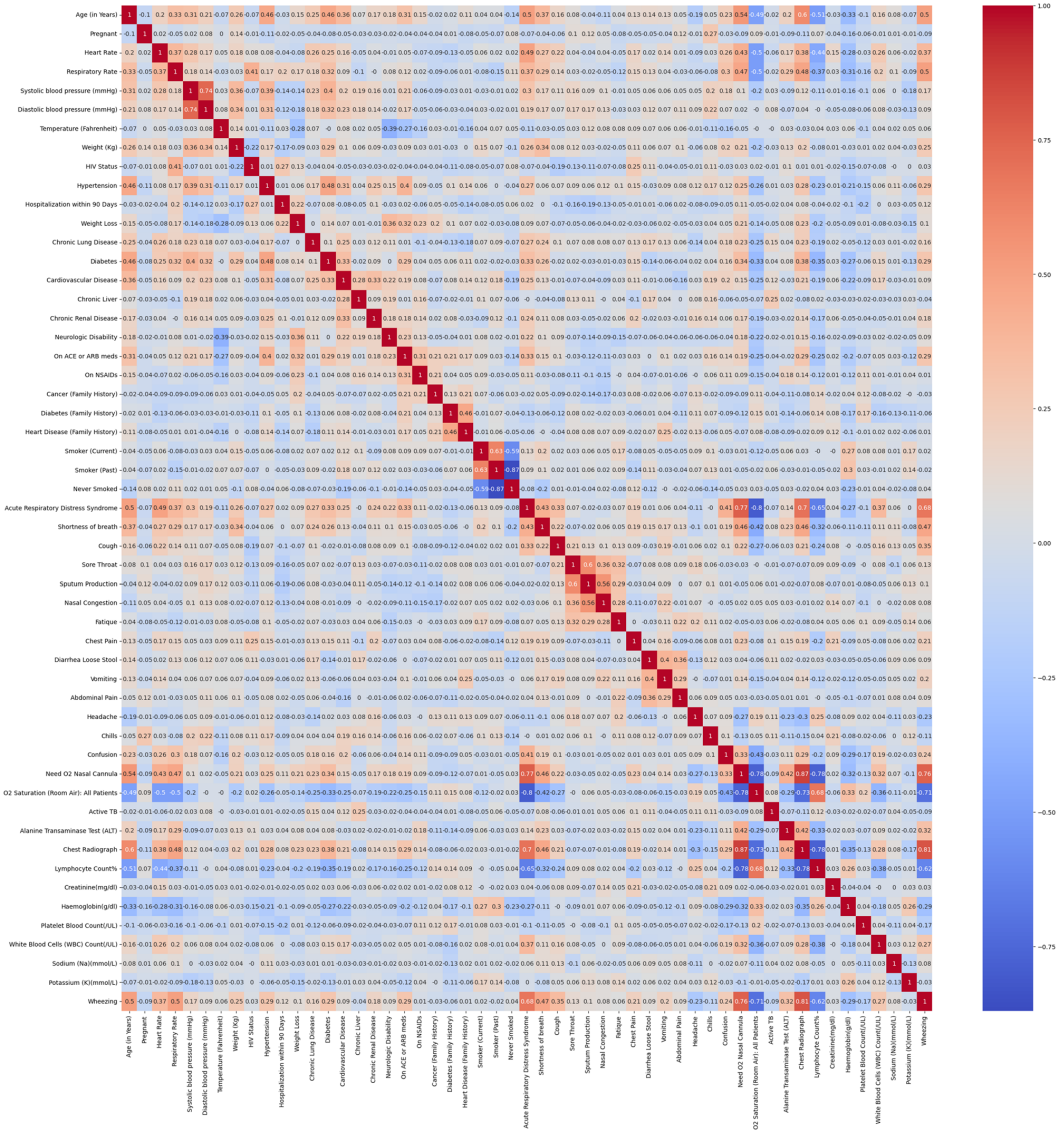
Pearson standard correlation coefficients for clinical features.

**Figure 3:**
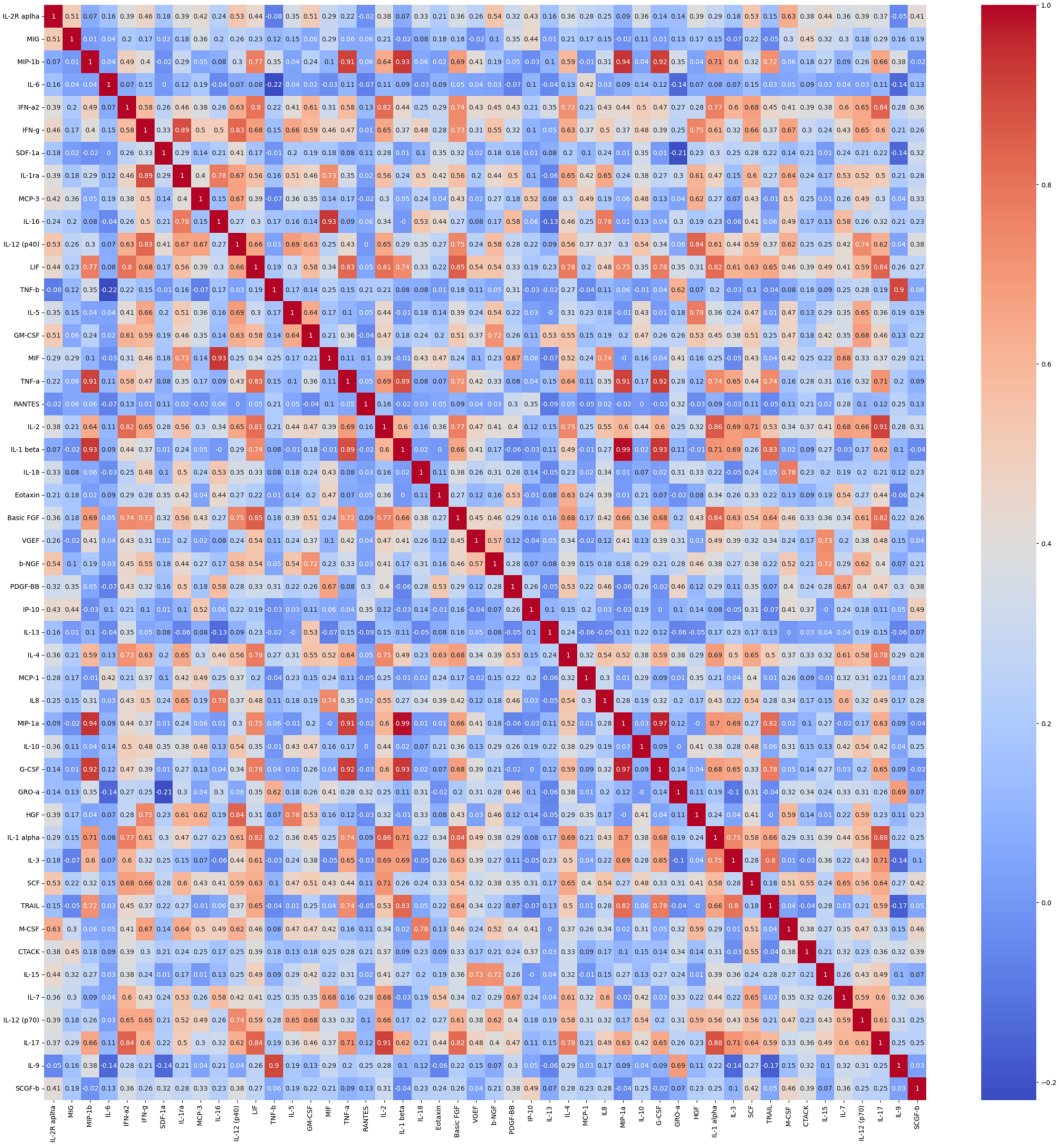
Pearson standard correlation coefficients for cytokine/chemokine features.

Machine Intelligence Learning Optimizer (MILO), an automated machine learning software for generating optimized binary classifiers was subsequently applied to these datasets^20,21^. MILO-ML utilized Python, Pandas, Scikit Learn, and ReactJS/VueJS, with additional custom libraries. The tool is available for academic use and evaluation at MILO-ML.com. The platform enables the end-to-end application of integrated ML methods, starting with initial data processing (scalers, etc.) and transformation (e.g., principal component analysis), feature selection and extraction (e.g., ANOVA F select percentile feature selection) and extending to model generation, validation, testing and deployment^19–21,26^. The MILO platform further applies custom hyperparameter combinations to generate models utilizing a diverse set of algorithms (k-nearest neighbors (k-NN), logistic regression (LR), Naïve Bayes (NB), random forest (RF), support vector machine (SVM), MLP neural network (NN), and XGBoost gradient boosting machine (GBM)). The dependent variable, depending on the method, either provides a binary outcome or a probability that is then converted into a binary outcome by using a threshold of 0.5 (with a possible range of 0 to 1). These algorithms and hyperparameter permutations are simultaneously evaluated within the MILO workflow to provide parallel generation of greater than 300,000 models from more than 1000 distinct pipelines (each defined by permutations of scalers, scorers, feature selectors, hyperparameter searchers and algorithms). This high-throughput workflow enables simultaneous and empiric assessment of a large search space to efficiently identify optimal models utilizing a range of performance metrics (accuracy, F1, sensitivity, specificity, PPV, NPV, etc.), based on the model performance on a secondary (generalization) dataset^26^. A training and initial validation test set with cross validation included a dataset of 150 cases (Dataset A: COVID-19 positive n = 75, COVID-19 negative n = 75) with 50 core clinical and laboratory parameters as well as 48 cytokines and chemokines (Pearson correlations seen in Figs. 2 and 3).

### Model interpretability

The methods utilized in this paper have well-defined transparent methodologies, as discussed in earlier works^19^. While our work was augmented with an automated machine learning tool to rapidly explore a diverse array of models, the techniques employed are statistical methods that have been used and studied for decades. The Neural Network approach is the only model utilized that contains an element of a “black box” in which the inner workings are not easily explainable beyond the layers, connections, and feedback between the various neurons. However, unlike a system like OpenAI’s Chat GPT, the foundational data and individual features being employed are all known and accessible for review or critique.

### Supplementary Information

Supplementary Information.

## Data Availability

The data is available upon reasonable request from Hooman H. Rashidi (corresponding author). Please make requests to rashidihh@upmc.edu.

## Abbreviations

NN: Neural network
LR: Logistic regression
NB: Naive Bayes
k-NN: K-nearest neighbors
SVM: Support vector machine
RF: Random Forest
XGBoost: Extreme gradient boosting
