# Supplementary material for: Comparing machine learning screening approaches using clinical data and cytokine profiles for COVID-19 in resource-limited and resource-abundant settings
Source: Sci Rep. 2024 Jun 28;14:14892. doi: 10.1038/s41598-024-63707-3 (PMC11211475; doi:10.1038/s41598-024-63707-3)
Supplement: Supplementary file 1 — Supplementary Information. [file 41598_2024_63707_MOESM1_ESM.pdf]

SUPPLEMENTAL MATERIALS

Supplemental Figure 1: ROC AUC/Precision Recalls Curves for Approaches 1 and 2

ROC AUC curves for the top models from Model Approach 1 (Clinical and Common Lab Data) and Approach 2 (Cytokine and Chemokine Profile) are shown below.

Approach 1: Clinical Lab Data

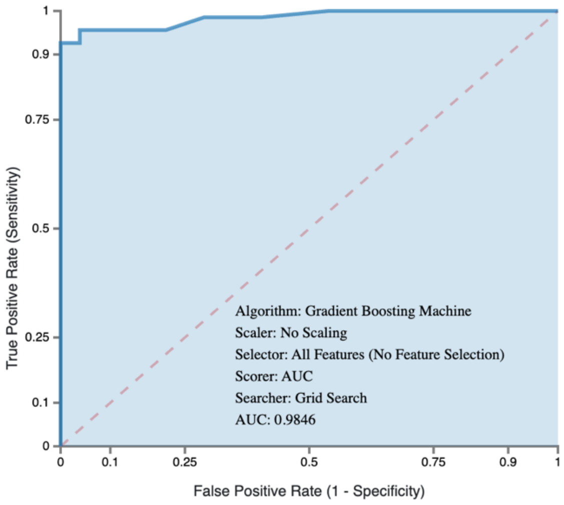

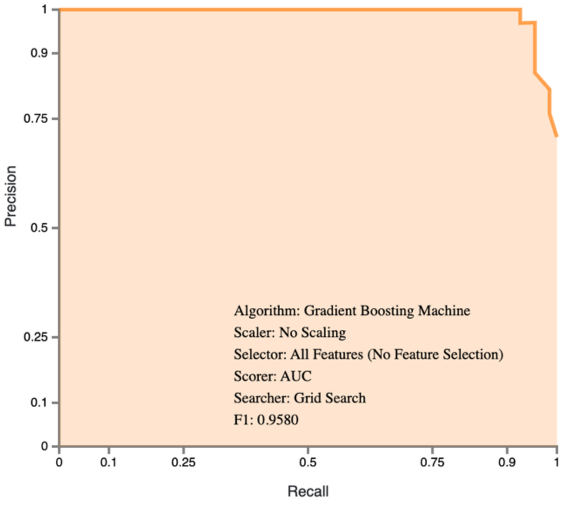

Approach 2: Cytokine Data

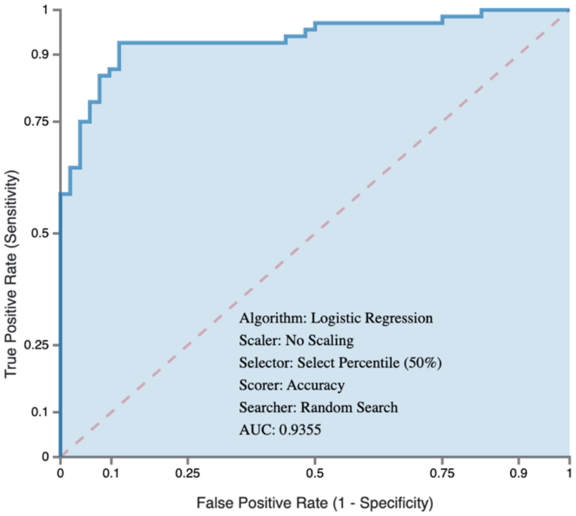

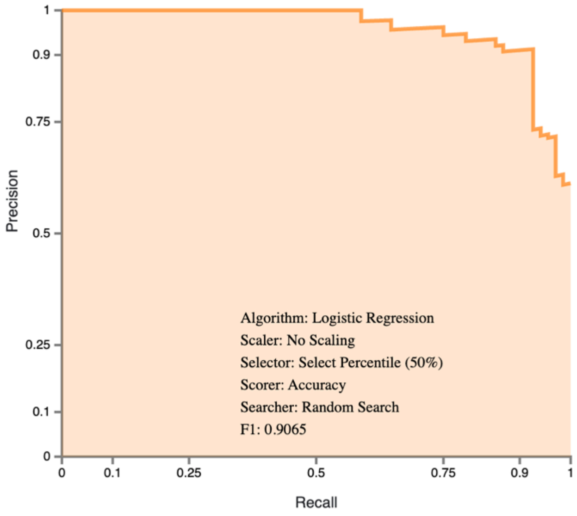

Supplemental Table 1: Additional Models for Approaches 1-3

Top Models Generated by MILO. (Sen. = sensitivity, Spec. = specificity)

Approach 1

Algorithm	Feature Selector	Scaler	Searcher	Scorer	Sen.	Spec.	ROC/AUC	Accuracy	Training ROC/AUC	PPV	NPV
gradient boosting machine	all features	no scaling	grid search	AUC	0.927	1.000	0.985	0.958	1.000	1.000	0.912
gradient boosting machine	all features	no scaling	random search	accuracy	0.927	1.000	0.988	0.958	1.000	1.000	0.912
gradient boosting machine	all features	no scaling	random search	AUC	0.927	1.000	0.988	0.958	1.000	1.000	0.912
gradient boosting machine	all features	no scaling	random search	F1	0.927	1.000	0.988	0.958	1.000	1.000	0.912
gradient boosting machine	random forest importance (25%)	no scaling	grid search	accuracy	0.927	1.000	0.985	0.958	1.000	1.000	0.912
gradient boosting machine	random forest importance (50%)	no scaling	grid search	accuracy	0.927	1.000	0.985	0.958	1.000	1.000	0.912
gradient boosting machine	random forest importance (50%)	no scaling	grid search	F1	0.927	1.000	0.985	0.958	1.000	1.000	0.912
gradient boosting machine	random forest importance (75%)	no scaling	2nd random search	AUC	0.941	0.981	0.985	0.958	1.000	0.985	0.927
gradient boosting machine	all features	min max scaler	grid search	accuracy	0.927	1.000	0.985	0.958	1.000	1.000	0.912
gradient boosting machine	all features	min max scaler	2nd random search	accuracy	0.941	0.981	0.986	0.958	1.000	0.985	0.927
gradient boosting machine	all features	min max scaler	2nd random search	accuracy	0.941	0.981	0.986	0.958	1.000	0.985	0.927
gradient boosting machine	all features	min max scaler	2nd random search	F1	0.941	0.981	0.986	0.958	1.000	0.985	0.927
gradient boosting machine	all features	min max scaler	2nd random search	F1	0.941	0.981	0.986	0.958	1.000	0.985	0.927
gradient boosting machine	random forest importance (50%)	min max scaler	grid search	accuracy	0.927	1.000	0.985	0.958	1.000	1.000	0.912
gradient boosting machine	random forest importance (50%)	min max scaler	grid search	F1	0.927	1.000	0.985	0.958	1.000	1.000	0.912

gradient boosting machine	random forest importance (75%)	min max scaler	grid search	F1	0.927	1.000	0.985	0.958	1.000	1.000	0.912
gradient boosting machine	all features	standard scaler	grid search	accuracy	0.927	1.000	0.985	0.958	1.000	1.000	0.912
gradient boosting machine	random forest importance (25%)	standard scaler	grid search	accuracy	0.927	1.000	0.985	0.958	1.000	1.000	0.912
gradient boosting machine	random forest importance (25%)	standard scaler	grid search	F1	0.927	1.000	0.985	0.958	1.000	1.000	0.912
gradient boosting machine	random forest importance (50%)	standard scaler	grid search	accuracy	0.927	1.000	0.985	0.958	1.000	1.000	0.912
gradient boosting machine	random forest importance (50%)	standard scaler	grid search	AUC	0.941	0.981	0.986	0.958	1.000	0.985	0.927

Approach 2

Algorithm	Feature Selector	Scaler	Searcher	Scorer	Sen.	Spec.	ROC AUC	Accuracy	Training ROC/AUC	PPV	NPV
logistic regression	select percentile (50%)	no scaling	2nd random search	F1	0.927	0.885	0.936	0.908	0.996	0.913	0.902
logistic regression	select percentile (50%)	no scaling	random search	accuracy	0.927	0.885	0.936	0.908	0.996	0.913	0.902
logistic regression	select percentile (50%)	no scaling	random search	F1	0.927	0.885	0.936	0.908	0.996	0.913	0.902
logistic regression	random forest importance (50%)	standard scaler	random search	AUC	0.912	0.885	0.963	0.900	0.969	0.912	0.885
logistic regression	select percentile (50%)	no scaling	2nd random search	AUC	0.927	0.865	0.934	0.900	0.996	0.900	0.900
logistic regression	select percentile (50%)	no scaling	2nd random search	accuracy	0.927	0.865	0.931	0.900	0.996	0.900	0.900
logistic regression	all features	no scaling	random search	accuracy	0.941	0.827	0.948	0.892	0.996	0.877	0.915
logistic regression	random forest importance (75%)	standard scaler	grid search	accuracy	0.897	0.885	0.947	0.892	1.000	0.910	0.868
logistic regression	random forest importance (75%)	standard scaler	grid search	accuracy	0.897	0.885	0.947	0.892	1.000	0.910	0.868
logistic regression	select percentile (75%)	no scaling	random search	F1	0.927	0.846	0.943	0.892	0.996	0.887	0.898

logistic regression	all features	no scaling	random search	AUC	0.941	0.827	0.940	0.892	0.907	0.877	0.915
logistic regression	random forest importanc e (75%)	no scaling	2nd random search	accuracy	0.927	0.846	0.939	0.892	0.996	0.887	0.898
logistic regression	random forest importanc e (75%)	no scaling	2nd random search	AUC	0.927	0.846	0.939	0.892	0.996	0.887	0.898
logistic regression	select percentile (75%)	no scaling	2nd random search	accuracy	0.927	0.846	0.938	0.892	0.996	0.887	0.898
logistic regression	select percentile (75%)	no scaling	2nd random search	F1	0.927	0.846	0.938	0.892	0.996	0.887	0.898
logistic regression	random forest importanc e (75%)	no scaling	2nd random search	AUC	0.927	0.846	0.936	0.892	0.991	0.887	0.898
logistic regression	select percentile (50%)	no scaling	2nd random search	AUC	0.912	0.865	0.934	0.892	0.996	0.899	0.882
logistic regression	select percentile (50%)	no scaling	grid search	accuracy	0.912	0.865	0.933	0.892	0.996	0.899	0.882
logistic regression	select percentile (50%)	no scaling	grid search	F1	0.912	0.865	0.933	0.892	0.996	0.899	0.882
logistic regression	select percentile (50%)	no scaling	grid search	AUC	0.912	0.865	0.930	0.892	0.996	0.899	0.882
logistic regression	select percentile (50%)	no scaling	random search	AUC	0.927	0.846	0.930	0.892	0.987	0.887	0.898

Approach 3

Algorithm	Feature Selector	Scaler	Searcher	Scorer	Sen.	Spec.	ROC AUC	Accuracy	Training ROC/AUC	PPV	NPV
gradient boosting machine	random forest importanc e (25%)	no scaling	grid search	AUC	0.956	0.981	0.970	0.967	1.000	0.985	0.944
gradient boosting machine	random forest importanc e (25%)	no scaling	grid search	AUC	0.956	0.981	0.970	0.967	1.000	0.985	0.944
gradient boosting machine	all features	no scaling	grid search	accuracy	0.927	1.000	0.985	0.958	1.000	1.000	0.912
gradient boosting machine	all features	no scaling	grid search	F1	0.927	1.000	0.985	0.958	1.000	1.000	0.912
gradient boosting machine	random forest importanc e (25%)	no scaling	grid search	accuracy	0.927	1.000	0.967	0.958	1.000	1.000	0.912
gradient boosting machine	random forest	no scaling	grid search	accuracy	0.927	1.000	0.968	0.958	1.000	1.000	0.912

	importance (25%)										
gradient boosting machine	random forest importance (25%)	no scaling	grid search	F1	0.927	1.000	0.967	0.958	1.000	1.000	0.912
gradient boosting machine	random forest importance (25%)	no scaling	grid search	F1	0.927	1.000	0.968	0.958	1.000	1.000	0.912
gradient boosting machine	random forest importance (25%)	no scaling	random search	accuracy	0.941	0.981	0.972	0.958	1.000	0.985	0.927
gradient boosting machine	random forest importance (25%)	no scaling	random search	F1	0.941	0.981	0.972	0.958	1.000	0.985	0.927
gradient boosting machine	random forest importance (50%)	no scaling	grid search	accuracy	0.927	1.000	0.985	0.958	1.000	1.000	0.912
gradient boosting machine	random forest importance (50%)	no scaling	grid search	accuracy	0.927	1.000	0.984	0.958	1.000	1.000	0.912
gradient boosting machine	random forest importance (50%)	no scaling	grid search	AUC	0.941	0.981	0.984	0.958	1.000	0.985	0.927
gradient boosting machine	random forest importance (50%)	no scaling	grid search	F1	0.927	1.000	0.985	0.958	1.000	1.000	0.912
gradient boosting machine	random forest importance (50%)	no scaling	2nd random search	accuracy	0.941	0.981	0.988	0.958	1.000	0.985	0.927
gradient boosting machine	random forest importance (50%)	no scaling	2nd random search	F1	0.941	0.981	0.988	0.958	1.000	0.985	0.927
gradient boosting machine	random forest importance (75%)	no scaling	grid search	accuracy	0.927	1.000	0.985	0.958	1.000	1.000	0.912
gradient boosting machine	random forest importance (75%)	no scaling	grid search	F1	0.927	1.000	0.985	0.958	1.000	1.000	0.912
gradient boosting machine	random forest importance (75%)	no scaling	random search	accuracy	0.941	0.981	0.988	0.958	1.000	0.985	0.927
gradient boosting machine	random forest importance (75%)	no scaling	random search	F1	0.941	0.981	0.988	0.958	1.000	0.985	0.927
gradient boosting machine	random forest importance (75%)	no scaling	2nd random search	accuracy	0.941	0.981	0.986	0.958	1.000	0.985	0.927

Supplemental Table 2: Descriptive Statistics for Dataset A (Training and Initial Validation, n=150)

	count	mean	std	min	25%	50%	75%	max
Age (in Years)	150	45.093	16.897	17	31.25	44.5	58	81
Pregnant	150	0.013	0.115	0	0	0	0	1
Heart Rate	150	88.747	20.528	18	76	86	98	152
Respiratory Rate	150	20.66	7.827	11	16	18	24	80
Systolic blood pressure (mmHg)	150	129.36	22.623	60	120	130	140	202
Diastolic blood pressure (mmHg)	150	80.513	14.704	30	70	80	90	130
Temperature (Fahrenheit)	150	98.185	11.481	1	98	99	101	104
Weight (Kg)	150	74.18	15.694	40	61.25	75	85	110
HIV Status	150	0.013	0.115	0	0	0	0	1
Hypertension	150	0.473	0.501	0	0	0	1	1
Hospitalization within 90 Days	150	0.04	0.197	0	0	0	0	1
Weight Loss	150	0.133	0.341	0	0	0	0	1
Chronic Lung Disease	150	0.1	0.301	0	0	0	0	1
Diabetes	150	0.327	0.471	0	0	0	1	1
Cardiovascular Disease	150	0.14	0.348	0	0	0	0	1
Chronic Liver	150	0.047	0.212	0	0	0	0	1
Chronic Renal Disease	150	0.053	0.225	0	0	0	0	1
Neurologic Disability	150	0.02	0.14	0	0	0	0	1
On ACE or ARB meds	150	0.127	0.334	0	0	0	0	1
On NSAIDs	150	0.087	0.282	0	0	0	0	1
Cancer (Family History)	150	0.1	0.301	0	0	0	0	1
Diabetes (Family History)	150	0.467	0.501	0	0	0	1	1
Heart Disease (Family History)	150	0.313	0.465	0	0	0	1	1
Smoker (Current)	150	0.133	0.341	0	0	0	0	1
Smoker (Past)	150	0.28	0.451	0	0	0	1	1
Never Smoked	150	0.693	0.463	0	0	1	1	1

Acute Respiratory Distress Syndrome	150	0.293	0.457	0	0	0	1	1
Shortness of breath	150	0.66	0.475	0	0	1	1	1
Cough	150	0.727	0.447	0	0	1	1	1
Sore Throat	150	0.56	0.498	0	0	1	1	1
Sputum Production	150	0.473	0.501	0	0	0	1	1
Nasal Congestion	150	0.293	0.457	0	0	0	1	1
Fatique	150	0.773	0.42	0	1	1	1	1
Chest Pain	150	0.18	0.385	0	0	0	0	1
Diarrhea Loose Stool	150	0.153	0.362	0	0	0	0	1
Vomiting	150	0.087	0.282	0	0	0	0	1
Abdominal Pain	150	0.14	0.348	0	0	0	0	1
Headache	150	0.54	0.5	0	0	1	1	1
Chills	150	0.16	0.368	0	0	0	0	1
Confusion	150	0.067	0.25	0	0	0	0	1
Need O2 Nasal Cannula	150	0.393	0.49	0	0	0	1	1
O2 Saturation (Room Air): All Patients	150	88.767	13.435	42	84	96	98	99
Active TB	150	0.013	0.115	0	0	0	0	1
Alanine Transaminase Test (ALT)	150	47.553	42.274	9	20	37.5	58	304
Chest Radiograph	150	0.46	0.5	0	0	0	1	1
Lymphocyte Count%	150	25.429	13.968	0.7	13.325	28	37	56
Creatinine(mg/dl)	150	1.008	1.532	0.1	0.7	0.8	0.995	19
Haemoglobin(g/dl)	150	13.566	1.969	7.7	12.2	13.55	15.1	18.4
Platelet Blood Count(/UL)	150	237.195	97.156	2.2	176	225	280.75	610
White Blood Cells (WBC) Count(/UL)	150	10.457	7.491	1.6	6.6	8.6	11.475	71
Sodium (Na)(mmol/L)	150	136.662	11.6	14	135	137	140	164
Potassium (K)(mmol/L)	150	4.436	0.673	2.46	4.002	4.5	4.8	7
Wheezing	150	0.373	0.485	0	0	0	1	1
*HGNC alternative names provided at base of table								

IL-2R alpha	150	118.96	71.234	29	71.25	97.5	151	492
MIG	150	753.82	1939.696	51	145	234	585.5	20485
MIP-1b	150	303.353	160.993	116	269	291	321.5	2129
IL-6	150	52.28	261.184	0	1	3	16.5	3041
IFN-a2	150	7.44	6.59	1	3	6	9	53
IFN-g	150	19.767	30.691	1	3.25	11	21.75	259
SDF-1a	150	1359.393	459.692	501	1035.5	1295.5	1622	3006
IL-1ra	150	1462.287	2243.436	52	312.25	699.5	1478.5	14528
MCP-3	150	9.167	16.089	0	1	3	8.75	99
IL-16	150	452.24	821.535	25	75.25	182.5	480.25	5450
IL-12 (p40)	150	111.607	124.03	15	45.25	82	127.75	1085
LIF	150	80.867	60.049	23	49.25	71	92.75	619
TNF-b	150	1857.547	364.288	554	1665.75	1857.5	2101.75	2650
IL-5	150	18.153	62.458	1	7	7	7	679
GM-CSF	150	6.607	5.525	0	3	5	8	43
MIF	150	4395.293	7145.692	184	812	2570	5102	52384
TNF-a	150	69.927	77.17	33	48.25	56	72	903
RANTES	150	22030.51	31352.41	876	8038.25	15709	23939.5	305593
IL-2	150	2.707	5.373	0	1	1	1	42
IL-1 beta	150	34.927	323.931	1	4	6	10	3974
IL-18	150	581.513	821.346	10	120	270.5	761.75	6974
Eotaxin	150	81.947	56.423	13	49	71	93.5	397
Basic FGF	150	25.967	20.37	3	15	23	31.75	187
VGEF	150	26.407	87.718	1	6	6	6	608
b-NGF	150	7.447	14.761	0	0	2	8	104
PDGF-BB	150	2013.167	1357.142	231	1120.75	1771	2392.25	9018
IP-10	150	2397.193	4951.196	13	261.5	512	1961.75	32947
IL-13	150	5.453	3.778	1	3	5	7	32
IL-4	150	5.593	2.761	3	4	5	6	22
MCP-1	150	55.053	112.235	2	11.25	20	50	845
IL8	150	164.247	567.883	1	10	22	57.25	4520
MIP-1a	150	14.527	131.278	0	1	2	3	1598
IL-10	150	10.28	13.706	0	4	7	12	127
G-CSF	150	284.767	1107.381	37	88.75	118	205	12791
GRO-a	150	1098.84	431.233	12	871.5	1146	1423	2239
HGF	150	2027.647	6575.35	186	419.75	574.5	1191.75	71624
IL-1 alpha	150	17.133	20.988	2	5	12	21.75	197
IL-3	150	0.233	0.923	0	0	0	0	8
SCF	150	93.787	50.983	35	61	78	108.5	328
TRAIL	150	48.24	57.219	1	27.25	39	48	621

M-CSF	150	58.487	45.192	7	26.25	44	73.75	278
CTACK	150	592.38	332.846	83	368.5	527	790.5	1884
IL-15	150	27.42	35.872	19	19	19	19	264
IL-7	150	10.38	19.491	2	3	3	3	140
IL-12 (p70)	150	4.253	5.205	0	2	2	4	35
IL-17	150	10.813	13.085	1	5	8	13	109
IL-9	150	688.527	154.145	241	641.25	710	772.75	1002
SCGF-b	150	90721.29	44385.86	14502	60229.25	79131.5	111085.8	251693
Covid-Status-Combined	150	0.5	0.502	0	0	0.5	1	1

* FGF basic (FGF2), Eotaxin (CCL11), G-CSF (CSF3), GM-CSF (CSF2), IFN-γ (IFNG), IL-1β (IL1B), IL-1ra (IL1RN), IL-1α (IL1A), IL-2Rα (IL2RA), IL3, IL-12 (p40) (IL12B), IL16, IL2, IL4, IL5, IL6, IL7, IL8 (CXCL8), IL9, GRO-α (CXCL1), HGF, IFN-α2 (IFNA2), LIF, MCP-3 (CCL7), IL10, IL-12 (p70) (IL12A), IL13, IL15, IL17A, IP-10 (CXCL10), MCP-1 (MCAF) (CCL2), MIG (CXCL9), β-NGF (NGF), SCF (KITLG), SCGF-β (CLEC11A), SDF-1α (CXCL12), MIP-1α (CCL3), MIP-1β (CCL4), PDGF-BB (PDGFB), RANTES (CCL5), TNF-α, VEGF (VEGFA), CTACK (CCL27), MIF, TRAIL (TNFSF10), IL-18 (IL18), M-CSF (CSF1), TNF-β (LTA).

Supplemental Table 3: Descriptive Statistics for Dataset B (Generalization Testing, n=120)

	count	mean	std	min	25%	50%	75%	max
Age (in Years)	120	48.525	19.112	17	30	47.5	65	86
Pregnant	120	0.008	0.091	0	0	0	0	1
Heart Rate	120	87.725	18.536	34	76.5	86	101	130
Respiratory Rate	120	21.033	7.756	11	16	19	23.25	58
Systolic blood pressure (mmHg)	120	125.875	21.874	51	110	130	140	212
Diastolic blood pressure (mmHg)	120	78.533	14.601	20	70	80	90	110
Temperature (Fahrenheit)	120	104.41	82.637	1	98	100	101	987
Weight (Kg)	120	73.817	16.376	40	62	71	84.25	128
HIV Status	120	0	0	0	0	0	0	0
Hypertension	120	0.492	0.502	0	0	0	1	1
Hospitalization within 90 Days	120	0.05	0.219	0	0	0	0	1
Weight Loss	120	0.117	0.322	0	0	0	0	1
Chronic Lung Disease	120	0.125	0.332	0	0	0	0	1
Diabetes	120	0.333	0.473	0	0	0	1	1
Cardiovascular Disease	120	0.192	0.395	0	0	0	0	1
Chronic Liver	120	0.058	0.235	0	0	0	0	1
Chronic Renal Disease	120	0.083	0.278	0	0	0	0	1

Neurologic Disability	120	0.025	0.157	0	0	0	0	1
On ACE or ARB meds	120	0.15	0.359	0	0	0	0	1
On NSAIDs	120	0.083	0.278	0	0	0	0	1
Cancer (Family History)	120	0.117	0.322	0	0	0	0	1
Diabetes (Family History)	120	0.383	0.488	0	0	0	1	1
Heart Disease (Family History)	120	0.292	0.456	0	0	0	1	1
Smoker (Current)	120	0.175	0.382	0	0	0	0	1
Smoker (Past)	120	0.267	0.444	0	0	0	1	1
Never Smoked	120	0.683	0.467	0	0	1	1	1
Acute Respiratory Distress Syndrome	120	0.325	0.47	0	0	0	1	1
Shortness of breath	120	0.717	0.453	0	0	1	1	1
Cough	120	0.775	0.419	0	1	1	1	1
Sore Throat	120	0.558	0.499	0	0	1	1	1
Sputum Production	120	0.4	0.492	0	0	0	1	1
Nasal Congestion	120	0.25	0.435	0	0	0	0.25	1
Fatique	120	0.725	0.448	0	0	1	1	1
Chest Pain	120	0.233	0.425	0	0	0	0	1
Diarrhea Loose Stool	120	0.15	0.359	0	0	0	0	1
Vomiting	120	0.1	0.301	0	0	0	0	1
Abdominal Pain	120	0.167	0.374	0	0	0	0	1
Headache	120	0.5	0.502	0	0	0.5	1	1
Chills	120	0.208	0.408	0	0	0	0	1
Confusion	120	0.167	0.374	0	0	0	0	1
Need O2 Nasal Cannula	120	0.458	0.5	0	0	0	1	1
O2 Saturation (Room Air): All Patients	120	86.633	15.183	35	80	93	98	99
Active TB	120	0.008	0.091	0	0	0	0	1
Alanine Transaminase Test (ALT)	120	42.636	45.572	6	18.75	29.5	47.25	258.3
Chest Radiograph	120	0.525	0.501	0	0	1	1	1
Lymphocyte Count%	120	29.519	80.613	1.069	9.35	21.05	33.25	891
Creatinine(mg/dl)	120	1.536	2.122	0.32	0.708	0.9	1.2	15.3

Haemoglobin(g/dl)	120	14.175	12.034	7.7	11.9	13.5	14.5	143
Platelet Blood Count(/UL)	120	236.273	90.259	44	176	236	280	525
White Blood Cells (WBC) Count(/UL)	120	10.015	5.194	3.4	6.75	8.5	11.235	34
Sodium (Na)(mmol/L)	120	136.004	13.473	0	135	137	140	150
Potassium (K)(mmol/L)	120	4.448	0.81	0	4.1	4.4	4.9	6.2
Wheezing	120	0.45	0.5	0	0	0	1	1
*HGNC alternative names provided at base of table								
IL-2R alpha	120	129.117	95.519	40	69.75	96.5	155.75	720
MIG	120	723.408	1378.148	37	133.25	236.5	660.5	9732
MIP-1b	120	279.867	81.797	38	240	279.5	314.25	786
IL-6	120	115.408	573.019	0	1	3	14.5	5823
IFN-a2	120	7.033	3.653	1	4	6	9	21
IFN-g	120	27.392	46.97	1	6	12	25	288
SDF-1a	120	1375.2	586.091	380	962.75	1262.5	1693	3495
IL-1ra	120	1917.55	3722.235	92	394	793.5	1602.5	30036
MCP-3	120	11.592	23.807	0	1	3	10.25	146
IL-16	120	449.375	739.647	22	86.5	243.5	422.25	6323
IL-12 (p40)	120	113.608	101.438	15	52.75	83.5	138	691
LIF	120	78.325	37.403	22	54	72	96.25	266
TNF-b	120	1748.25	434.859	260	1511.25	1777	2036.5	2679
IL-5	120	10.417	18.288	1	7	7	7	143
GM-CSF	120	5.867	3.557	1	4	5	7	22
MIF	120	3327.05	4455.378	355	961.25	2152	4382.5	42901
TNF-a	120	67.317	63.381	16	46.75	56	65	606
RANTES	120	18867.17	20630.27	640	7767.25	14970	23180.25	161535
IL-2	120	1.875	2.569	0	1	1	1	21
IL-1 beta	120	7.342	5.449	2	4	6	9	33
IL-18	120	725.258	994.128	18	121.5	362	886.75	6009
Eotaxin	120	87.392	66.749	21	43.75	67.5	110.75	373
Basic FGF	120	29	22.078	4	18	25	34.25	205
VGEF	120	11.108	30.144	1	6	6	6	300
b-NGF	120	6.567	9.712	0	0.75	3.5	7	54
PDGF-BB	120	2055.858	1962.932	180	1051.25	1558	2783.5	17941
IP-10	120	2755.992	5339.063	48	287	748	2793.25	40824
IL-13	120	5.5	3.435	1	3	5	7	21

IL-4	120	5.308	1.873	2	4	5	6	13
MCP-1	120	130.558	437.442	3	11	18.5	39.75	3798
IL8	120	96.092	359.511	1	10	23	48.75	3528
MIP-1a	120	5.233	20.883	1	1	2	3	208
IL-10	120	16.45	60.818	1	4.75	7	12.25	605
G-CSF	120	357.158	1543.681	39	93.5	123	181	16156
GRO-a	120	990.908	509.878	24	649	1059.5	1307.75	3447
HGF	120	2071.342	4504.441	139	416.5	701	1301.25	40205
IL-1 alpha	120	19.458	31.033	2	7	15	22	327
IL-3	120	0.233	0.683	0	0	0	0	4
SCF	120	97.875	65.474	20	60	82.5	110.5	459
TRAIL	120	42.1	32.743	1	26	36	50	213
M-CSF	120	67.808	56.613	7	30	50	89	310
CTACK	120	628.617	400.6	66	374.25	542	783	2893
IL-15	120	19.567	4.895	19	19	19	19	70
IL-7	120	7.267	13.031	2	3	3	4	95
IL-12 (p70)	120	2.75	2.19	0	2	2	3	12
IL-17	120	10.208	7.023	1	6	9	13	41
IL-9	120	648.342	174.651	66	570.75	685.5	767.25	946
SCGF-b	120	94666.96	50309.64	31720	61866.75	82741	110549.5	357503
Covid-Status-Combined	120	0.567	0.498	0	0	1	1	1

* FGF basic (FGF2), Eotaxin (CCL11), G-CSF (CSF3), GM-CSF (CSF2), IFN-γ (IFNG), IL-1β (IL1B), IL-1ra (IL1RN), IL-1α (IL1A), IL-2Rα (IL2RA), IL3, IL-12 (p40) (IL12B), IL16, IL2, IL4, IL5, IL6, IL7, IL8 (CXCL8), IL9, GRO-α (CXCL1), HGF, IFN-α2 (IFNA2), LIF, MCP-3 (CCL7), IL10, IL-12 (p70) (IL12A), IL13, IL15, IL17A, IP-10 (CXCL10), MCP-1 (MCAF) (CCL2), MIG (CXCL9), β-NGF (NGF), SCF (KITLG), SCGF-β (CLEC11A), SDF-1α (CXCL12), MIP-1α (CCL3), MIP-1β (CCL4), PDGF-BB (PDGFB), RANTES (CCL5), TNF-α, VEGF (VEGFA), CTACK (CCL27), MIF, TRAIL (TNFSF10), IL-18 (IL18), M-CSF (CSF1), TNF-β (LTA).
